# Effect of ethanol and cocaine on [^11^C]MPC-6827 uptake in SH-SY5Y cells

**DOI:** 10.1007/s11033-021-06336-7

**Published:** 2021-04-20

**Authors:** Naresh Damuka, Miranda Orr, Paul W. Czoty, Jeffrey L. Weiner, Thomas J. Martin, Michael A. Nader, Avinash H. Bansode, Buddhika S. Liyana Pathirannahel, Akiva Mintz, Shannon L. Macauley, Suzanne Craft, Kiran Kumar Solingapuram Sai

**Affiliations:** 1 Department of Radiology, Wake Forest School of Medicine, Winston-Salem, NC 27157 USA; 2 Department of Internal Medicine, Wake Forest School of Medicine, Winston-Salem, NC USA; 3 Department of Physiology and Pharmacology, Wake Forest School of Medicine, Winston-Salem, NC USA; 4 Department of Anesthesiology, Wake Forest School of Medicine, Winston-Salem, NC USA; 5 Department of Radiology, Columbia University Medical Center, New York, NY USA

**Keywords:** Microtubule, Cytoskeleton, Substance use disorder, In vitro binding, Biomarkers

## Abstract

Microtubules (MTs) are structural units in the cytoskeleton. In brain cells they are responsible for axonal transport, information processing, and signaling mechanisms. Proper function of these processes is critical for healthy brain functions. Alcohol and substance use disorders (AUD/SUDs) affects the function and organization of MTs in the brain, making them a potential neuroimaging marker to study the resulting impairment of overall neurobehavioral and cognitive processes. Our lab reported the first brain-penetrant MT-tracking Positron Emission Tomography (PET) ligand [^11^C]MPC-6827 and demonstrated its in vivo utility in rodents and non-human primates. To further explore the in vivo imaging potential of [^11^C]MPC-6827, we need to investigate its mechanism of action. Here, we report preliminary in vitro binding results in SH-SY5Y neuroblastoma cells exposed to ethanol (EtOH) or cocaine in combination with multiple agents that alter MT stability. EtOH and cocaine treatments increased MT stability and decreased free tubulin monomers. Our initial cell-binding assay demonstrated that [^11^C]MPC-6827 may have high affinity to free/unbound tubulin units. Consistent with this mechanism of action, we observed lower [^11^C]MPC-6827 uptake in SH-SY5Y cells after EtOH and cocaine treatments (e.g., fewer free tubulin units). We are currently performing in vivo PET imaging and ex vivo biodistribution studies in rodent and nonhuman primate models of AUD and SUDs and Alzheimer's disease.

## Introduction

Cytoskeletal defects, including alterations in microtubule stability, axonal transport and actin dynamics, have been characterized in several psychiatric and neurodegenerative disorders, including alcohol and substance use disorders (AUD/SUDs) and Alzheimer’s disease, suggesting they are a common feature contributing to neurodegeneration. An organized neuronal cytoskeleton is required for nervous system development, maintenance, and regenerative processes after injury. Its three components intermediate filaments, actin filaments, and microtubules (MTs) or tubulins, all play a vital role in neurological processes. MTs are critical to cellular structure; as neuronal backbones they facilitate cell division, axonal transport, and neurotransmission. MTs are hetero-dimer units formed from α- and β-tubulin monomers [1]. Essential biophysical functions, including cellular signaling and axoplasmic transport, depend on the structural integrity of MTs i.e., polymerization with bound and free tubulin units and MT integrity is heavily dysregulated in AUD and SUDs [2–5]. Addictive behaviors lead to many adaptations in postsynaptic spine structure that result in profound alterations in synaptic transmission [6]. At the molecular level, synaptic activity triggers diverse signaling pathways, which, in turn regulate and reorganize cytoskeleton-associated proteins. For example, repeated cocaine administration has been shown to change stathmin, a regulatory protein crucial to MT dynamics [6, 7], causing morphologic changes [8].

Neuronal structural changes may contribute to the progression of AUD and SUDs [9]. Repeated doses of 5–20 mM or 0.08 g/dL of alcohol and 0.3–0.5 mg/kg of cocaine in humans and non-human primates respectively were considered to be interfering with normal brain functions [10–13]. Chronic ethanol (EtOH) exposure significantly stabilizes neuronal and acetylated MTs in hepatic PC12 cells [14], increases dendrite lengths and neurite outgrowth and causes aberrant sprouting of hippocampal neuritis [15]. Loss of α and β free tubulin units in the caudate nucleus, cortex, and cerebellum was noted in post-mortem brain samples from individuals diagnosed with AUD. Repeated exposure to drugs of abuse like EtOH and cocaine induces structural plasticity [14, 16] in many brain circuits and changes in the density and morphology of dendritic spines [5, 17, 18]. These alterations have significant consequences including cognitive deficits and neurodegeneration [19, 20]. Prolonged SUD is also associated with brain injury characterized by impaired synaptogenesis, cellular migration, and neurogenesis—all of which require proper MT functioning [3, 21]. MT agents (MTAs), believed to work primarily by altering MT network integrity, are widely being investigated as drug candidates to treat cancer, brain disorders, and cardiovascular diseases. Thus, MT integrity is important to many neurochemical pathways commonly associated with AUD and SUD. However, studies of cytoskeleton-dependent structural plasticity resulting from EtOH and cocaine use have focused predominantly on actin and filament dynamics; molecular level MT impairments remains largely unexplored. Positron emission tomography (PET) imaging is a sensitive modality to examine and quantify in vivo MT-based changes in the neurochemical cascades of SUD.

MPC-6827 is a small molecule MTA that causes mitotic arrest and cell death. It exerts antitumor (glioblastoma) properties by binding to β-tubulin sites. We reported the automated radiochemical synthesis of [^11^C]MPC-6827 as the first brain-penetrating, MT-tracking PET ligand and imaged it in vivo in normal rodents and non-human primates [22, 23]. To establish the potential of [^11^C]MPC-6827 as a PET imaging ligand for various neurological disorders, we need to investigate its mechanism of action. Here, we report our preliminary in vitro evaluations of [^11^C]MPC-6827 in SH-SY5Y neuroblastoma cells [24–26] with (a) two different abused drugs (EtOH and cocaine), and (b) various MT stabilizing and destabilizing agents.

## Methods

**Fig. 1:**
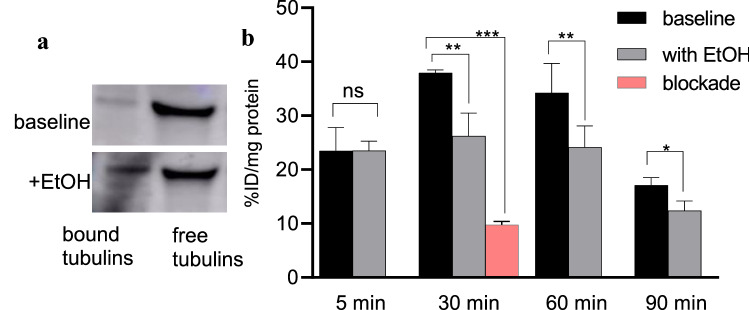
Representative bound and free tubulins, **a** western blots and **b** [^11^C]MPC-6827 uptake in vitro at 5, 30, 60 and 90 min incubation times in SH-SY5Y cells with EtOH (100 mM/3 days) and without EtOH treatment (n = 6/group); *p ≤ 0.05, **p ≤ 0.021, ***p ≤ 0.0016, and ns: non-significant

**Fig. 2:**
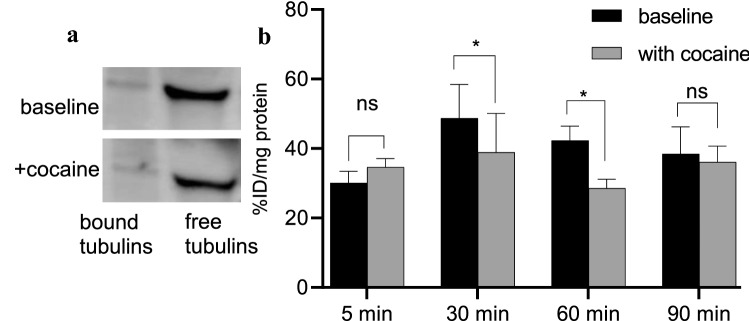
Representative bound and free tubulins, **a** western blots and **b** [^11^C]MPC-6827 uptake in vitro at 5, 30, 60 and 90 min incubation times in SH-SY5Y cells with cocaine (1 mM/3 days) and without cocaine treatment (n = 6); *p < 0.05 and ns: non-significant

To investigate the effects of EtOH and cocaine on tubulin dynamics, we performed a MT-based assay (Cytoskeleton, Inc., Denver, CO, USA) [27–29] in SH-SY5Y neuroblastoma cells treated with 100 mM EtOH [14] and 1 mM cocaine [8] (n = 6/group) respectively for 3 days. We chose this concentration as it does not affect cell viability and neurites growth. This commercially available kit separates large complexes of polymerized tubulins/MTs attached to nuclei and Golgi bodies into bound and non-polymerized free tubulins. After ultra-centrifugation, supernatant and pellet portions with high free- and bound-tubulins respectively, were loaded on SDS-PAGE for western blot analyses. An enhanced chemiluminescence kit was used to visualize the tubulin bands [14, 15], (Figs. 1a and 2a). Bound/stabilized tubulin content was significantly higher and unbound/free α/β tubulins lower in EtOH-treated cells than control cells treated with PBS. Cells treated with cocaine showed no significant difference in bound tubulins and slightly fewer free α/β tubulins than untreated cells possibly due to the accrued rate of MT polymerization with substances. Therefore, both EtOH and cocaine compromise MT integrity i.e., increase in bound and decrease in free tubulin units. Having demonstrated these drug-induced changes in MT integrity in SH-SY5Y cells, we next aimed to determine whether [^11^C]MPC-6827 could also detect similar MT alterations in the same cells.

We performed cell binding assays in vitro in SH-SY5Y cells with [^11^C]MPC-6827 following our previously published protocols.[30–32] The cells (0.25–5.0 × 10^6^ cells/well) were treated with 100 mM EtOH or 1 mM cocaine [8] (n = 6/group) for 3 days. We then measured radiotracer binding by adding [^11^C]MPC-6827 (1–2 µCi/0.12–0.24 nM/well) and incubating the cells for 5, 30, 60, and 90 min at room temperature (n = 6/time point). To demonstrate tracer specificity, a subgroup of cells (n = 3) was pre-treated with non-radioactive MPC-6827 (1.0 µM), adding radiotracer 60 min later and incubating for 30 min. To demonstrate tracer sensitivity to length of drug exposure, cells were treated with 100 mM EtOH or 1 mM cocaine for 1 h, 1 day, or 3 days and incubated with [^11^C]MPC-6827 for 30 min at room temperature All the cells were then washed with PBS and lysed with 1 N NaOH. Finally, the lysate from each well was γ-counted (PerkinElmer, Waltham, MA, USA) and counts-per-minute (cpm) values were normalized to the amount of radioactivity added to each well. The uptake data in each sample from each well and the standard counts for each condition were expressed as cpm of activity and were decay corrected for elapsed time. Using the CPM values, the protein concentration in the well was calculated as percent uptake relative to the control condition i.e., expressed as % injected dose (ID)/mg of protein present in each well, with p values ≤ 0.05 considered statistically significant.

## Results and discussion

**Fig. 3:**
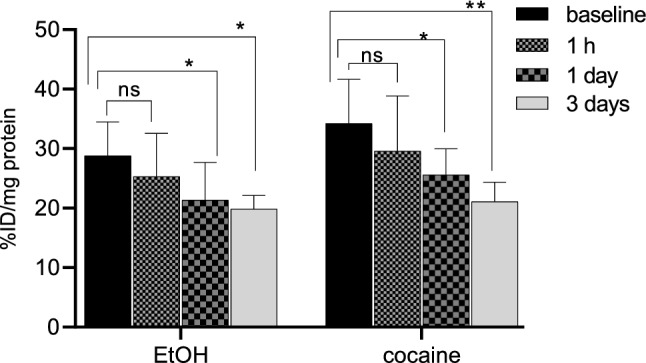
[^11^C]MPC-6827 uptake in vitro with EtOH (100 mM) and cocaine (1 mM) for 1 h, 1 day and 3 day exposures in SH-SY5Y cells (n = 6); *p ≤ 0.05, **p ≤ 0.019, and ns: non-significant

EtOH- (Fig. 1b) and cocaine-treated (Fig. 2b) cells demonstrated an ~ 30(± 2) and ~ 24(± 6) percent decrease respectively in radioactive uptake versus non-treated controls over the 30–90 min incubation times. Additionally, uptake in EtOH-treated and cocaine-treated cells increased ~ 13(± 3) and ~ 12(± 2) percent from 5 to 30 min of incubation times respectively and decreased ~ 53(± 2) and ~ 19(± 3) percent by 90 min in EtOH- and cocaine-treated cells; thus demonstrating favorable pharmacokinetics. For the self-blocking assays (Fig. 1b), uptake was ~ 78(± 1) percent lower after addition of nonradioactive MPC-6827, demonstrating high specificity. Some nonspecific binding was always associated with radiotracer evaluations in PET imaging [33, 34] and likely due to in vitro artifacts and/or not completely saturating the target site. These assays were primarily used to demonstrate a proof-of-principle for in vitro specificity. Baseline uptake with ethanol and cocaine was slightly different probably due to varied amount of cells in each well and/or specific activity of [^11^C]MPC-6827 on the day of experiment. Radioactive uptake was decreased ~ 21(± 1) and 28(± 1) percent from 1 h to 3 days EtOH and cocaine exposures (Fig. 3) respectively. Therefore, [^11^C]MPC-6827 uptake decreased with increased exposure to EtOH or cocaine. Moreover, since no significant decrease in radioactivity was observed after 3 days of drug exposure we used the same 3 days exposure in all our assays. MPC-6827 primarily targets the β tubulin site at pharmacological doses [35–37]. The lowered radioactive uptake in EtOH- and cocaine-treated SH-SY5Y cells indicates that [^11^C]MPC-6827 uptake correlate well with observed bound/free tubulin changes and may be tracking free β tubulin units, as both substance treatments decreased free tubulin content in the same cells.

**Fig. 4:**
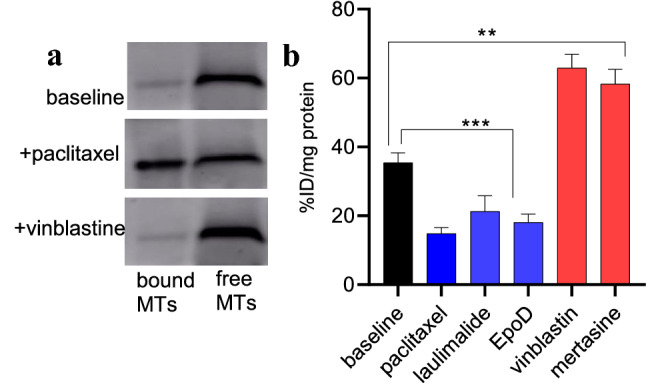
Representative bound and free tubulin, a. western blots and b. [^11^C]MPC-6827 uptake in vitro with different MT agents (1 µM) for 180 min) in SH-SY5Y cells (n = 6); **p ≤ 0.011,***p ≤ 0.0015

MTAs are categorized as either stabilizing agents (paclitaxel, laulimalide, and EpoD) [38–40], which favor polymerization of tubulin units and inhibit cell proliferation, or destabilizing agents (vinblastine and mertasine) [41–44], which increase free/unbound tubulins and promote apoptotic cell death. To distinguish their effect on MT integrity in SH-SY5Y cells, we performed the same tubulin-based western blot assays on paclitaxel- and vinblastine-treated cells [45]. The paclitaxel-treated cells had more bound/stabilized tubulins, and vinblastine-treated cells had more unbound/free tubulins than the untreated cells (Fig. 4a). To confirm the free tubulin-based binding mechanism of [^11^C]MPC-6827, SH-SY5Y cells were pretreated with different MTAs at 1.0 µM concentration, 3.0 h prior to addition of [^11^C]MPC-6827. Paclitaxel, laulimalide and EpoD decreased radioactive uptake by ~ 58(± 3), ~ 40(± 4), and ~ 66(± 7) percent respectively, while vinblastine, and mertasine increased it by ~ 77(± 6), and 64(± 5) percent respectively (Fig. 4b), confirming that [^11^C]MPC-6827 may primarily target free tubulin units.

## Conclusions

Results of the preliminary [^11^C]MPC-6827 in vitro assays with EtOH and cocaine treatments at different incubation times in SH-SY5Y cells indicate that radioactive uptake decreases with increased drug exposure. Tests with various MTAs demonstrate that [^11^C]MPC-6827 may preferentially bind to free/unbound tubulin units with high selectivity. The radioactive uptake results were well-corroborated with observed changes in bound and free tubulin expressions in SH-SY5Y cells with EtOH and cocaine treatments. Taken together, these studies confirm that [^11^C]MPC-6827 has great potential as an MT imaging agent for defining MT-based mechanisms that underlie the development of alcohol and cocaine addiction. We are currently characterizing its complete in vivo and ex vivo imaging properties in both rodent and nonhuman primate models of AUD/SUDs and Alzheimer's disease.

## Sources

Cocaine hydrochloride and other reagents were purchased from Sigma Aldrich; MPC 6827 hydrochloride was purchased from Tocris a biotech brand; SH-SY5Y cells was purchased from american type culture collection (ATCC) and the precursor for [^11^C]MPC-6827 was purchased from ABX Inc supplies.

## Data Availability

All the data and materials are available upon request.
